# Relationship between patient safety and accountability of nurses in Al-Zahra Gilangharb Hospital in 2015

**Published:** 2015

**Authors:** F Esfandnia, E Mohammadi, M Mohammadi, R Cheraghi, N Esfandnia, A Esfandnia

**Affiliations:** * Student Research Committee, Kermanshah University of Medical Sciences, Kermanshah, Iran

**Keywords:** patient safety, accountability, responsiveness, nurse, Gilangharb Hospital

## Abstract

**Introduction.** The purpose of this research was to investigate the connection between the patient safety and the accountability of nurses in Gilangharb Hospital in 2015.

**Methods.** This research was a cross-sectional study conducted in Al-Gilangharb, in 2015. The data needed for research was taken from the library and an internet search and was gathered by using standard questionnaireThe professional and caring nurses’ questionnaire was based on the extension evaluation office Nursing Care, Ministry of Health and Medical Education and demographic information and questions about 4 different roles of nurses were prepared and included. Moreover, patient safety was highlighted in a validation questionnaire, validated by experts judging group of teachers and academics, which was established. Cronbach’s alpha test was used to assess the reliability. Finally, the reliability and professional standards of nursing care, patient safety questionnaire 093. 86/ 0 percent calculated the population of Gilangharb Hospital nurses (n = 70) and, in the strata selected, a statistical analysis using data from the questionnaires included in the SPSS statistical software, version 21, took place.

**Results.** The patients’ safety and accountability was observed at the level of 95 percent by using the Spearman correlation (SIG = .000). The correlation coefficient was (R=.768). Also, the dimensions of responsibility between the patient’s safety (regarding the role of the nurse teacher, manager, researcher, and clinician) at 95 percent and the positive use of Spearman correlation was found (SIG = .000).

**Conclusions.** Given the correlation among the patient protection and accountability, it can be said that the nurses in all roles (educator, researcher, administrator, and clinical specialist) have been successful, so, we suggested that given the experience, expertise and abilities, they have made an efficient use of their lifting power.

## Introduction

Since the task and mission of critical health care and community life represent the quality of services in the health sector, it has a special place [**1**]. Patient safety is one of the principal parts of health care, in the sense of avoiding the introduction of any injury to the patient during health care [**2**] and includes items such as medication errors (wrong type or dose of medication prescribed), applying to compromise (acting in the wrong position, wrong technique, postoperative complications), false diagnosis (delayed diagnosis, misdiagnosis, incorrect diagnosis), failure of plant and equipment, leading to misdiagnosis and other cases, such as nosocomial infections, patient falls, bed sores, wrong treatment, etc. [**3**]. 

**The issue of patient safety (Patient safety)**

After the release of the report of the Institute of Medicine (Institute of Medicine-IOM), in the United States, in 1999, whose role was to discover the prevalence of medical errors in this country, it has been of interest to researchers and experts in the health field [**4**]. This report, together with reports of similar other institutions in countries like Britain, Canada and Australia, on this topic, made the international monitoring health regimes understand that they are not safe enough [**3**]. According to the available evidence, it was estimated that, in developing countries, one out of ten patients enters and appeals to hospital services while being injured. However, there are no accurate statistics on this issue in developing countries. The World Health Organization estimates that tens of millions of people are lost or suffer from disabilities every year due to medical errors and unsafe processes [**5**]. Care and treatment aids for people suffering from a heavy financial burden represent an unsafe increasing to the estimated time that would come forth, in fact between 5% and 10% of the health-related costs resulting from non-clinical help that is safe. The proportion of patients is represented by the damage and failure of systems and procedures over the role of individuals [**6**]. In addition, nursing, which is one of the biggest health care provider groups in the society and private health sectors, is more in contact with patients than nurses and other personnel providing care and performance and activity resulting from the cooperation of a joining of terms such as nurse, health, environment, and nursing [**6**]. In addition to the basic tasks related to the clinical care of patients (based on their professional mission delegation which means a lot of responsibility in different areas), professional nurses are responsible for eight general standards, the following indicators being considered: 1. accountability. The continuity of expertise and efficiency. The application of information, skills, and judgment. 4. Professional ethics. 5. Professional communication and participation. 6. Professional administration and supervision. 7. The quality of care. 8. Self-control and rating of performance. 

**Four major roles in determining the nursing profession.**

These roles include the clinical expert, educator, administrator, and researcher. One or more roles for nurses concern thelocation and the skill level [**7**]. Accountability represents the situation of the person in charge for the work of the others. Currently, professional accountability is very necessary for the age of accountability and rapid variations in the health system in which treatment occurs. As defined by the standard of accountability, “directing professional standards of nursing practice are determined based on the scope and range of performance”. Based on this definition, each nurse is responsible for her performance, guidance, and direction to her performance on the road to professional and legal standards. Also, the nurse should provide optimal clinical care and should be skilled and responsive to community. The World Health Organization identifies the significance of patient safety and public health as a main concern of the World Health Assembly resolution - WHO 55/ 18, and outlines the various responsibilities of the organization chart, which include technical support of Member States for the development of reporting systems and risk reduction, setting evidence-based policies, promoting a safety culture and encouraging research into patient safety [**8**]. Regarding the research in Iran, several problems were found in fields related to patient safety, including the incidence of bedsores, infection, and falling from bed. With regard to the importance of patient safety and a wide range of effects on patients and our health care system, and given that Employees in organizations in which resources are scarce and diverse skills and capabilities and flexibility have helped improve organizational performance is effective, The solutions and projects aimed at promoting changes in order to achieve greater safety for patients who need urgent health care system is growing, this study showed the importance of patient safety and accountability of nurses in hospitals in Al-Zahra Gilangharb. Daily, many patients in hospitals worldwide are safely treated and cured, but with the knowledge and technology in recent decades, health care facilities are more very complex. Naturally, in such circumstances, the probability of increased risk in health care facilities and experimental evidence indicates that the count of patients who are due to complications of medical errors is indispensable. Therefore, the issue of patient safety is a crucial issue in the health systems of different countries [**7**]. With regard to the importance of patient safety, a wide range of effects has been taken on patients and our health care system. According to that, staff resources are scarce and if you put the skills and capabilities of a variety and flexibility to the organization’s competing advantage, the enhancement of organizational achievement is of an effective help, according to the role of human resources in service organizations, especially hospitals and the importance of the operation level of the organization, this study investigating the way patient safety and accountability of nurses are explored. In a research undergone in 2011, Movahedgar and Arabs tried to identify the perceptions of patients in clinical departments of general hospitals of Tehran University of Medical Sciences to participate in treatment decisions and patient safety showing that the abnormal signals that might be interpreted by a person were probably explained differently by another person. This variety was due to demographic differences. The patient participation in treatment decisions affected his assessment of the protection of the patient in the hospital [**9**]. In 2011, Ravaghi and et al. described the connection between the perception of a culture of patient safety and care providers, patients’ perception of medical errors in public hospitals in Tehran showing that patients in hospitals, with a more positive safety culture, presented one of more errors experience and were providers of a more accurate understanding of some aspects of safety culture regarding the patient compared to other dimensions respectively. These dimensions included organizational knowledge and continuous development and cooperation within the unit [**1**]. During a study performed in 2011, Zh. Abdi demonstrated the culture of patient safety as the harvest workers in selected hospitals of Tehran Medical University showed that various aspects of safety culture in that hospital needed to improve and assess the culture of patient safety in hospitals, being able to assume multiple roles. According to a study by Moghry and et al. performed in on the one hand, there can be a strong or weak degree of safety culture of the center and it is clear on the other hand, that managers and supervisors have the potential to increase staff awareness of patient safety, contributing to improvement [**10**]. The Persian translation of a questionnaire validated patient protection culture surveys of hospital (HSOPSC) and the assessment of protection culture from the viewpoint of nurses, physicians, laboratory and radiology staff of public hospitals in Tehran University of Medical Sciences in Iran has confirmed validity and reliability to work [**11**]. In 1389, the doctor and his colleagues presented the attitudes regarding the safety of employees of a medical center, a hospital in Tehran, to evaluate the safety of a hospital staff showing that the attitude of supervisors and employees was comparatively low and efforts of promoting hospital safety culture were essential (Singer et al., 2009) [**12**]. to examine the connection between safety climate and safety performance in the hospital, 12 Patient safety indicators (PSIs) were presented and the study showed a meaningful connection between a safety climate and stronger and safer performance in hospitals [**13**]. In the research of Al-e Ahmad (2010), examining the connection between a safety climate hospital and hospital performance in patient safety indicators (PSIs), a meaningful connection was found between a safety climate and stronger and safer performance in hospitals [**14**]. Following this, in 2010, Screw et al. showed the connection between the culture of patient safety and the adverse events in US hospitals studied. In their study, from the connection between the 15 variables related to patient safety culture surveys of hospital and eight patient safety indicators for the expected (negative) and the connection of these 15, seven were statistically significant. In other words, a better protection culture in hospitals was associated with lower rates of opposing events [**2**]. In 2010, Homer and his colleagues titled psychometric characteristics of hospital care in the patient protection culture for hospital management (HSOPS_M) so as to investigate a wide range of sub-dimensions questionnaire HSOPS, such as feedback and conversation about errors/ events, organizational learning, hands and transferring personnel, and, their team work showed that with the help HSOPS_M, the survey of hospitals could be viewed as a cross-country evaluation of senior management on a protection culture at the hospital and as a measurement tool to support interventions in the hospital in terms of safety performance and the attitudes and perceptions of senior management, expectations, and Senior management of hospital patient safety measures on fundamental aspects of a safety culture [**15**]. 

**Main hypothesis:**

1) Are there any patient safety events regarding responsibility in Al Gilangharb? 

**Sub assumptions:**

1) Are there any safety events with an index clinician nurse regarding the relationship in Gilangharb Al-Zahra hospital? 

2) Are there any events in hospital regarding patient safety indicators or the nurse educator role in Gilangharb Al-Zahra? 

3) Are there any patient safety events concerning the role of the director of nursing in Gilangharb Al-Zahra hospital? 

4) Are there any researchers regarding the function of nurses in hospital patient safety events in Gilangharb Al-Zahra? 

## Methods 

This research was cross-sectional and was carried out in 2015 in Al Gilangharb. Data needed for research in using the library and internet exploration were conducted using standard questionnaires. According to the evaluation office extension of the Nursing Care Ministry of Health and Medical Education, the questionnaire professional nursing care was prepared and included demographic information and questions about 4 different nurses roles (clinician, educator, administrator, researcher) and patient safety was highlighted by Likert scale responses from very disagree to very agree with the grading of 1 to 5, respectively. The validation questionnaire validated by experts judged group of teachers and specialist academics that were established. To assess the reliability, Cronbach's alpha was applied. Finally, reliability and professional standards of nursing care, 86/ 0 was calculated and, given that the value of alpha was of more than 7.0, the questionnaire was desirable and acceptable. The group consisted of hospital nurses in Gilangharb (70 cases), and the Census Select the strata. Data from questionnaires were examined by using statistical software version SPSS 21.

## Results 

Based on demographic information, 58.6 percent were women and 41.4 percent were men. 63.2 percent and 36.8 percent were single, married, 50.1% between 25 and 30 years old, 19.7 Drsd 31-35 years, 12.5 percent Byn 36 and 40 years, 10.4% between 41 and 45 years and 7.3 percent more than 45 years old. 46.1 percent had a bachelor’s degree, 25.1% a high school degree, 11.7% had a higher education degree and 17 percent a MS degree. 

**Table 1:** Evaluation of data normality - Kolmogorov-Smirnov test

	safety	Accountability	Researcher	manager	Training Providers	Specialist Clinical	
N	385	385	385	385	385	385	
Normal Parameters a, b	Mean	196.6052	126.1221	14.8779	29.2416	37.4260	44.5766
	Std. Deviation	61.94874	31.88061	4.08671	8.09490	11.06752	12.77877
	Absolute	0.071	0.077	0.108	0.089	110	-0.106
Most Extreme Differences	Positive	0.071	0.077	0.105	0.089	110	0.106
	Negative	-0.069	-0.059	-0.108	-0.080	-0.054	-0.064
Kolmogorov-Smirnov Z	1.392	1.502	2.126	1.753	2.154	2.079	
Asymp. Sig. (2-tailed)	0.042	0.022	0.000	0.004	0.000	0.000	
a. Test distribution was Normal.							
b. Calculated from data.							

Based on the table above for normal data, One-Sample Kolmogorov-Smirnov Test was used as the significance level and less than 0.05 results showed that profits were not so normal data and Spearman test was used to examine the data.

**Table 2:** The relationship between the hypotheses using Spearman’s rho test

Correlations			safety
Spearman’s rho	Accountability	R	0.768
		SIG	0.000
		N	70
	Specialist Clinical	R	0.817
		SIG	0.000
		N	70
	Training Providers	R	0.622
		SIG	0.000
		N	70
	manager	R	0.691
		SIG	0.000
		N	70
	Researcher	R	0.666
		SIG	0.000
		N	70
			

The table earlier showed that patient safety and accountability was situated at the level of 95 percent, the use of Spearman correlation was positive (SIG = .000) regarding the correlation coefficient for the 768. The main hypothesis was confirmed. Between patient safety and accountability at the level of 95%, using Spearman and positive connection existed (SIG = .000) and secondary hypotheses were also approved. 

**Table 3:** Mean standard deviation and the mean rating by Friedman test to rank the dimensions of responsibility

	Mean	Std. Deviation	Mean Rank
Researcher	14.8779	4.08671	1.00
manager	29.2416	8.09490	2.09
Training Providers	37.4260	11.06752	3.09
Specialist Clinical	44.5766	12.77877	3.82

The above table displayed the largest average standard deviation for the later Specialist Clinical, the rate being 12.77877 ± 44.5766. The average standard deviation of Training Providers, manager, Researcher, was 4.08671 ± 14.8779 11.06752 ± 37.4260,8.09490 ± 29.2416 and also the results used the Friedman test with 1048.856 Chi-Square = And DF = 3 And SIG = .000 to rank the dimensions of the responsibility that the Specialist Clinical had with an average 3.82 rating and dimensions, Training Providers, manager, Researcher respectively, with an average rank of 3.09, 2.09 and 1, having the highest average rating. 

## Conclusion 

The results showed that a positive and important connection between patient safety and accountability could agree that the main theory was approved; patient safety and positive connection between the dimensions of responsibility were therefore sub-hypotheses that were confirmed. In 2012, Stoic et al. suggested that patients in hospitals with a positive safety culture would experience fewer errors [**1**]. Screw and colleagues showed that a better safety culture in hospitals was associated with lower rates of adverse events [**2**]. In 2011, Hope et al. indicated that safety programs had a positive impact on reducing accidents indicators that would play a role in decreasing the severity of accidents, reduce accident frequency index, reduce the severity of accidents and loss of a repeated diseases-frequency, various amounts of leading and indicated job consequently, increasing the level of productivity for any organization [**16**] particularly in the area of governance and leadership strategies using aggressive teeth, a hospital patient safety could be considered as a priority strategy and upgrade [**17**]. In 2014, Hemati Maslakpak stated that nurses could use the appropriate communication skills with patients in intensive care, maintain, and improve safety [**18**]. Friedman rank test results showed that nurses in roles of responsibility, such as Specialist Clinical, Training Providers, manager, and Researcher respectively, had the highest priority. Kim and colleagues demonstrated that the error reporting and coordination between sectors were identified as priority cases [**19**]. With consideration to the connection between patient protection and responsibility, it could be said that nurses in all roles (teacher, researcher, administrator and specialist clinical) have been successful, so we suggested that given the experience, expertise, and their ability and power, they could force the operation for an efficient use. In addition to the increase of patient safety and accountability, nurses need to attract collaborate and have contract with the employment from a treaty change and, according to the theory of equality of benefits, they are changed. 
